# Analysis of *Paracoccidioides* secreted proteins reveals fructose 1,6-bisphosphate aldolase as a plasminogen-binding protein

**DOI:** 10.1186/s12866-015-0393-9

**Published:** 2015-02-27

**Authors:** Edilânia Gomes Araújo Chaves, Simone Schneider Weber, Sonia Nair Báo, Luiz Augusto Pereira, Alexandre Melo Bailão, Clayton Luiz Borges, Célia Maria de Almeida Soares

**Affiliations:** Laboratório de Biologia Molecular, Instituto de Ciências Biológicas, ICBII, Campus II, Universidade Federal de Goiás, 74001-970 Goiânia, Goiás Brazil; Laboratório de Microscopia, Departamento de Biologia Celular, Instituto de Ciências Biológicas, Universidade de Brasília, Brasília, Distrito Federal Brazil

**Keywords:** *Paracoccidioides*, Proteome, Secretome, Plasminogen-binding proteins, Fructose 1,6-bisphosphate aldolase

## Abstract

**Background:**

Despite being important thermal dimorphic fungi causing Paracoccidioidomycosis, the pathogenic mechanisms that underlie the genus *Paracoccidioides* remain largely unknown. Microbial pathogens express molecules that can interact with human plasminogen, a protein from blood plasma, which presents fibrinolytic activity when activated into plasmin. Additionally, plasmin exhibits the ability of degrading extracellular matrix components, favoring the pathogen spread to deeper tissues. Previous work from our group demonstrated that *Paracoccidioides* presents enolase, as a protein able to bind and activate plasminogen, increasing the fibrinolytic activity of the pathogen, and the potential for adhesion and invasion of the fungus to host cells. By using proteomic analysis, we aimed to identify other proteins of *Paracoccidioides* with the ability of binding to plasminogen.

**Results:**

In the present study, we employed proteomic analysis of the secretome, in order to identify plasminogen-binding proteins of *Paracoccidioides*, *Pb*01. Fifteen proteins were present in the fungal secretome, presenting the ability to bind to plasminogen. Those proteins are probable targets of the fungus interaction with the host; thus, they could contribute to the invasiveness of the fungus. For validation tests, we selected the protein fructose 1,6-bisphosphate aldolase (FBA), described in other pathogens as a plasminogen-binding protein. The protein FBA at the fungus surface and the recombinant FBA (rFBA) bound human plasminogen and promoted its conversion to plasmin, potentially increasing the fibrinolytic capacity of the fungus, as demonstrated in fibrin degradation assays. The addition of rFBA or anti-rFBA antibodies was capable of reducing the interaction between macrophages and *Paracoccidioides*, possibly by blocking the binding sites for FBA. These data reveal the possible participation of the FBA in the processes of cell adhesion and tissue invasion/dissemination of *Paracoccidioides*.

**Conclusions:**

These data indicate that *Paracoccidioides* is a pathogen that has several plasminogen-binding proteins that likely play important roles in pathogen-host interaction. In this context, FBA is a protein that might be involved somehow in the processes of invasion and spread of the fungus during infection.

## Background

The *Paracoccidioides* genus comprises a complex of pathogenic fungi, classified in at least four distinct phylogenetic lineages: S1, PS2, PS3 and *Pb*01-like [1-3]. These fungi are thermally dimorphic, growing at room temperatures as mycelium, which produces infectious conidia. The inhalation of conidia or mycelia propagules by the human host and their differentiation to yeast cells initiates paracoccidioidomycosis (PCM), a major health problem in South America. This human systemic mycosis is considered the tenth leading cause of chronic disease mortality among infectious and parasitic diseases, and the first among the systemic mycoses in Brazil (51.2% of cases of deaths) [4-6].

Pathogenic microorganisms are able to penetrate and colonize host tissues by establishing complex interactions with the host molecules. Some microorganisms degrade extracellular matrix components (ECM) by using proteins that subvert proteases of the host itself [7-9]. Reports have shown that pathogens can capture plasminogen (Plg) and its activation could substantially augment the organism’s potential to tissue invasion and necrosis [10-20]. In eukaryotes, Plg is converted to its proteolytic form, plasmin, by physiological activators such as tissue type plasminogen activator (tPA) and urokinase type (uPA) [16]. Plasmin dissociates blood clots due to its role in the degradation of fibrin polymers and promotes the dissociation of the ECM components, which is relevant for dissemination of pathogens [17-22].

There is a variety of Plg-binding proteins and activation mechanisms used by pathogens. Besides the physiological activators, molecules produced by microorganisms, can also activate plasminogen. Studies describe various Plg-binding and activating proteins involved in the degradation of host tissues, components of ECM, which favors the spread and dissemination of different pathogens [14,23-25]. In bacteria, Plg-binding and activating proteins have been characterized [12-14,24,26-37]. Those proteins can increase the bacteria fibrinolytic activity, which favors tissue degradation and rapid progression of infection [35,38,39]. The importance of Plg in fungi is indicated by the Plg-binding properties of human pathogens, including *Candida albicans* [40,41], *Cryptococcus neoformans* [15], *Pneumocystis carinii* and *Aspergillus fumigatus* [42,43] that depict proteins at surface, which make them able to bind Plg, and improve ther infectiveness.

The high dissemination of *Paracoccidioides* spp. from the site of infection to different tissues, underscores the importance of understanding the fungi virulence factors and their effects in human host. In a previous study developed by our group, we reported the recruitment of Plg and its activation into plasmin, by *Paracoccidioides*, *Pb*01, through tPA, in a process mediated by the protein enolase [10]. The enolase of *Paracoccidioides* is a surface associated protein that promotes an increase in the adhesion and invasion of the fungus to host cells in *in vitro* models of infection [10,44,45]. The recombinant *Paracoccidioides* enolase is able to adhere to some ECM components and to the surface of macrophages, reinforcing the role of this molecule in the host-pathogen interaction [46]. These data highlight that Plg-binding proteins increase the potential for invasion and pathogenicity of *Paracoccidioides* through the fibrinolytic activity of plasmin. Proteins with this ability may be transported to the surface of the fungus and secreted into the external medium and promote plasmin formation, which also contributes to the pathogen dissemination [47]. In this sense, the enolase of *Paracoccidioides* is constitutively secreted by the yeast and mycelia phases [48], and is detected in the fungal cell wall [10].

In the present study, we employed proteomic analysis of the secretome, in order to identify Plg-binding proteins of *Paracoccidioides*, *Pb*01. Fifteen Plg-binding proteins were present in the fungal secretome. Proteins of the glycolytic pathway, such as phosphoglycerate kinase, glyceraldehyde-3-phosphate dehydrogenase and fructose 1,6-bisphosphate aldolase (FBA) were identified; the last was selected for further characterization. FBA has been described in various microorganisms as a Plg-binding protein, but its role has not been described in thermally dimorphic fungi. Here we show that *Paracoccidioides* binds Plg via FBA, that is found at the surface and secreted by the fungus. The protein binds human plasminogen (hPlg) and converts it into plasmin, in the presence of tPA. The interaction of the protein with hPlg, promoted increased fibrinolytic capacity of the fungus, as tested in fibrin degradation assays. The addition of recombinant FBA (rFBA) or anti-rFBA antibodies was capable of reducing the interaction between macrophages and *Paracoccidioides*, possibly by blocking the binding sites for FBA. These data reveal the possible participation of the FBA in the *Paracoccidioides* adhesion and invasion processes. The identification of novel surface/secreted proteins that can be involved in host-pathogen interaction is central to understand *Paracoccidioides* pathogenesis.

## Results and discussion

### Identification of plasminogen-binding proteins of *Paracoccidioides*, *Pb*01 yeast cells

**Figure 1:**
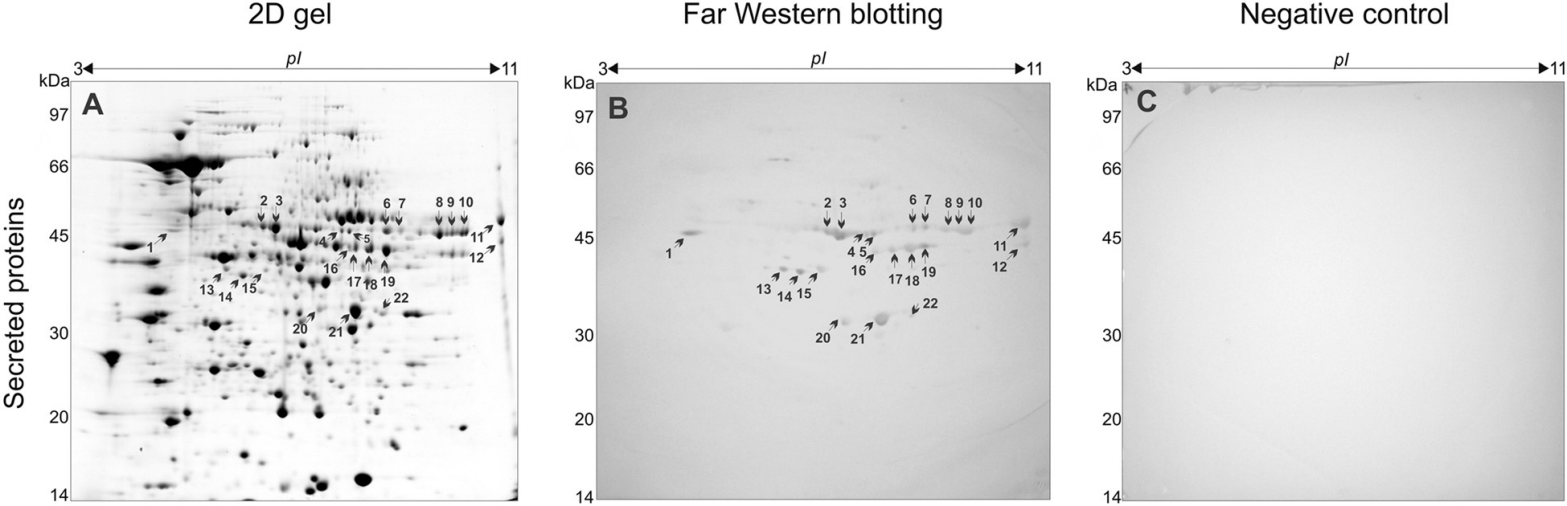
**Detection of plasminogen-binding proteins of** ***Paracoccidioides*** **,** ***Pb*** **01.** Secreted proteins (500 μg) of *Paracoccidioides, Pb*01, were subjected to 2-DE [first dimension: IEF pH range 3–11 non-linear, second dimension: 12% (w/v) SDS-PAGE)]. 2-DE gels were stained using Coomassie brilliant blue **(Panel A)** or transferred to nitrocellulose membranes that were processed for Far-western blotting **(Panel B)**. Negative controls include a membrane not incubated with Plg **(Panel C)**. Membrane in panel B was incubated with 35 μg/ml of hPlg, followed by the incubation with the anti-hPlg antibody (1 μg/mL) and the reaction was developed with BCIP/NBT. To identify in the 2-DE gel, the proteins spots that were visualized in the membrane, a pairing of proteins spots was performed, using Image master 2D Platinum software (GE Healthcare). The pH gradient is shown above the gel, and the molecular mass protein standards (kDa) are indicated to the left of the gels.

In order to identify Plg-binding proteins in the secretome of *Paracoccidioides, Pb*01, we obtained 2-DE gels. The gels ran in parallel, were (i) stained with Coomassie brilliant blue or (ii) transferred to nitrocellulose membrane and reacted with Plg, in a Far-western blotting assay, as demonstrated in Figure 1, panel B. Image analysis were produced allowing the pairing of the proteins spots between the 2-DE gel and the membrane obtained by Far-western blotting.

The detected spots in the membrane (Figure 1B) were compared to the Coomassie blue partners in order to find their corresponding proteins spots in the 2-DE gel (Figure 1A). Subsequently, protein spots were manually excised of the gel, and identified by mass spectrometry. It was possible to identify in the secretome of yeast cells, 22 protein spots, which bound Plg, as depicted in Figure 1B. Figure 1, panel C, depicts the images of the negative control assay, in which the membrane was not previously incubated with Plg, indicating no cross-reactivity of the proteins with the antibody to Plg.

Spots identified as Plg-binding proteins were cut from the gel and subjected to tryptic digestion and mass spectrometry analysis. The data were used to search the Mascot, and provided the identification of 15 proteins/isoforms. Table 1 describes the secreted proteins of *Paracoccidioides*, identified as Plg-binding molecules. Several enzymes were detected in this category, some of them presenting several isoforms, such as homogentisate 1,2-dioxygenase (spots 4,5), NADP-specific glutamate dehydrogenase (spots 6,7), phosphoglycerate kinase (spots 8,16) 2-methylcitrate synthase (spots 9,10,11), FBA (spots 13,14,15) and malate dehydrogenase (spots 20,21). Thus, the 22 protein spots identified are summed up in 15 different proteins.

**Table 1:** **Secreted proteins of** ***Paracoccidioides*** **that bind plasminogen**

**Spot number** ^**1**^	**General information number (NCBI)** ^**2**^	**Protein description**	***pI*** **theor/exp** ^**3**^	**MM (kDa) theor/exp** ^**4**^	**PMF score** ^**5**^	**Coverage sequence (%)** ^**6**^	**MS/MS Ions score** ^**7**^	**Matched peptides** ^**8**^	**Psort prediction** ^**9**^	**SignalP Value ≥ 0.5** ^**10**^	**SecretomeP Value ≥ 0.5** ^**11**^	**big-PI** ^**12**^
**1**	gi|226285916	aminomethyl transferase	9.67/4.42	53.35/45.56	121	37	84	4	mito: 23.0	NO	0.516	NO
**2**	gi|226278634	aldehyde dehydrogenase	5.92/6.94	54.69/45.97	94	59	114	6	cyto: 21.5	NO	0.562	NO
**3**	gi|295668479	formamidase	6.10/7.13	46.14/45.71	144	44	109	5	cyto: 12.0	NO	0.565	NO
**4**	gi|295658700	homogentisate 1,2-dioxygenase	6.25/7.62	50.85/45.51	78	35	-	-	cyto: 13.0	NO	0.601	NO
**5**	gi|295658700	homogentisate 1,2-dioxygenase	6.16/7.76	50.86/45.35	76	26	89	4	cyto: 13.0	NO	0.621	NO
**6**	gi|295659664	NADP-specific glutamate dehydrogenase	7.66/8.48	50.38/45.35	102	53	72	4	cyto: 11.0	NO	NO	NO
**7**	gi|295659664	NADP-specific glutamate dehydrogenase	7.17/8.75	50.46/45.45	101	56	117	2	cyto: 11.0	NO	NO	NO
**8**	gi|295669690	phosphoglycerate kinase	6.48/9.49	45.31/ 44.54	83	61	151	5	cyto: 25.0	NO	NO	NO
**9**	gi|295666179	2-methylcitrate synthase	9.02/9.73	51.51/45.00	-	-	226	6	mito: 27.0	NO	NO	NO
**10**	gi|295666179	2-methylcitrate synthase	9.02/9.96	51.51/ 44.92	95	62	95	4	mito: 27.0	NO	NO	NO
**11**	gi|295666179	2-methylcitrate synthase	9.02/10.66	51.58/47.76	78	57	88	4	mito: 27.0	NO	NO	NO
**12**	gi|295658119	glyceraldehyde-3-phosphate dehydrogenase	10.18/10.67	33.92/43.65	-	-	93	2	cyto: 27.0	NO	0.532	NO
**13**	gi|295671120	fructose 1,6-bisphosphate aldolase	6.09/6.41	39.72/41.29	-	-	154	5	cyto: 21.0	NO	0.505	NO
**14**	gi|295671120	fructose 1,6-bisphosphate aldolase	6.09/6.60	39.72/41.29	-	-	670	5	cyto: 21.0	NO	0.505	NO
**15**	gi|295671120	fructose 1,6-bisphosphate aldolase	6.09/6.88	39.72/40.94	-	-	555	9	cyto: 21.0	NO	0.505	NO
**16**	gi|295669690	phosphoglycerate kinase	6.48/7.75	45.31/42.67	86	59	56	3	cyto: 25.0	NO	NO	NO
**17**	gi|295668707	acetyl-CoA acetyltransferase	8.98/7.88	46.65/42.67	-	-	102	3	mito: 24.5	NO	0.692	NO
**18**	gi|11496183	immunodominant antigen Gp43	7.17/8.15	45.77/42.42	97	43	102	4	extr: 24.0	NO	0.746	NO
**19**	gi|226285552	ketol-acid reductoisomerase	9.12/8.46	44.86/42.17	172	62	134	7	mito: 27.0	NO	0.683	NO
**20**	gi|295658218	malate dehydrogenase	6.36/7.18	34.67/33.98	73	47	69	5	cyto: 17.0	NO	0.674	NO
**21**	gi|295658218	malate dehydrogenase	6.36/7.85	34.67/33.75	129	41	344	9	cyto: 17.0	NO	0.674	NO
**22**	gi|226279168	2,5-diketo-D-gluconic acid reductase A	7.71/8.40	34.78/33.36	81	48	50	3	cyto: 20.5	0.5	NO	NO

^1^Spots numbers indicated in Figure 1A.

^2^NCBI database general information number (http://www.ncbi.nlm.nih.gov/).

^3^Isoelectric point (theoretical/experimental).

^4^Molecular Mass in kDa (theoretical/experimental);

^5^Mascot PMF score for fragmentation data (http://www.matrixscience.com).

^6^Sequence coverage percentage.

^7^Mascot MS/MS score for fragmentation data (http://www.matrixscience.com).

^8^Number of identified peptides (MS/MS).

^9^Subcellular localization prediction of proteins according Psort (http://www.genscript.com/psort/wolf_psort.html).

^10^Secretion prediction according to Signal P 3.0 server. The number corresponds to signal peptide probability (Score³ 0.5) (http://www.cbs.dtu.dk/services/SignalP/).

^11^Secretion prediction according to Secretome P 1.0 server; the number corresponds to neural network that exceeded a value of 0.5 (NN-score ³ 0.50) (http://www.cbs.dtu.dk/services/SecretomeP/).

^12^GPI Modification Site Prediction of proteins according big-PI (http://mendel.imp.ac.at/gpi/gpi_server.html).

cyto: cytoplasm.

extr: extracellular.

mito: mitochondria.

While much of the proteins described in this work are not annotated in the database Psort (http://www.genscript.com/psort/wolf_psort.html) as extracellular proteins, we found compatible data in other studies. The proteins: 2-methylcitrate synthase, FBA, glyceraldehyde 3-phosphate dehydrogenase, formamidase, acetyl-CoA acetyltransferase and phosphoglycerate kinase were detected in the secretome of *Paracoccidioides, Pb*01 yeast and mycelia [48]. Other proteins were identified in the secretome of *Paracoccidioides, Pb*18: FBA, glyceraldehyde 3-phosphate dehydrogenase and phosphoglycerate kinase [49]. These data corroborate the *in silico* analysis performed in the software Signal P and Secretome P, where we can observe that most of the proteins described here are secreted by nonclassical pathways (Table 1).

Some of the proteins identified in this study have also been described in other systems as Plg-binding proteins. In this way, acetyl-CoA acetyltransferase was identified in the bacteria *Leptospira interrogans* [50]; phosphoglycerate kinase was described in *C. albicans* [40], *Streptococcus pneumoniae* [51], as well as in *C. neoformans* [15]. In addition, FBA and glyceraldehyde 3-phosphate dehydrogenase were also described as Plg-binding proteins in *C. albicans* [40].

Formamidase is a highly abundant protein in *Paracoccidioides*, as previously described by our group [52,53]. The protein gp43 also detected in our binding assays, binds to laminin, putatively contributing to the fungus virulence and facilitating the process of infection [54,55].

The proteomic binding assays, also allowed the identification of enolase as a Plg-binding protein. The presence of glycolytic enzymes as Plg-binding proteins is reported in several pathogens, including bacteria and fungi. In *Paracoccidioides*, enolase is present at the yeast cells surface, where it binds and activates hPlg, presumably contributing to the fungus pathogenesis [10]. Other glycolytic enzymes, such as glyceraldehyde 3-phosphate dehydrogenase, phosphoglycerate kinase and FBA, were found here as Plg-binding proteins (Figure 1B, Table 1). Glyceraldehyde 3-phosphate dehydrogenase (GAPDH), is a molecule that binds Plg and is present on the surface and secretome of bacteria [56-58] and fungi [40]. In *C. albicans*, this molecule is an adhesin that participates in the process of adherence to human cells, and binds to ECM components [40,59-61]. In studies conducted by our group, GAPDH is located at the surface of *Paracoccidioides*, where could mediate the adhesion and internalization of the fungus to host cells, binding to ECM components [62].

Phosphoglycerate kinase is an adhesin in both, bacteria [63] and fungi [15,40]. On the surface of group B streptococcus, phosphoglycerate kinase binds the host actin and Plg. Binding of ECM components to bacterial proteins, including phosphoglycerate kinase, promotes the activation of specific proteins on its surface, which induces bacterial adhesion [63,64]. Also, proteolytic degradation of ECM by phosphoglycerate kinase - recruited plasmin activity, promotes adherence to endothelial cells and bacterial dissemination in the host tissues [36]. In *C. neoformans*, phosphoglycerate kinase localizes to the fungal cell wall, where exhibits accessible carboxyl-terminal lysine residues for Plg-binding [65].

FBA is cytoplasmic and also localized at the surface of several bacteria [66,67], as well as in pathogenic fungi [15,40] where it binds host molecules and depicts adhesin function, beyond its glycolytic activity. In this work, three isoforms of FBA were detected (Table 1, spots 13, 14 e 15). The FBA of *Paracoccidioides*, *Pb*01 was previously characterized in our laboratory [68,69]. The protein is as an antigenic molecule, reactive with sera of PCM patients, as demonstrated [68]. Studies revealed the role of FBA in cell adhesion and invasion [67]. The FBA-deficient mutant of *Neisseria meningitides* was not affected in its ability to grow *in vitro*, but depicted a significant reduction in adhesion to human brain microvascular endothelial and HEp-2 cells, suggesting participation in adhesion of meningococci to human cells [67]. In *C. neoformans*, analysis of the Plg-binding proteins, allowed the identification of a FBA surface protein, that serves as a Plg receptor [15]. So, due to the relevance of FBA as an adhesin and a Plg-binding protein that promotes the virulence of microorganisms, the protein was selected for further investigation in *Paracoccidioides.*

### Confirmatory assays of FBA as a plasminogen-binding protein

**Figure 2:**
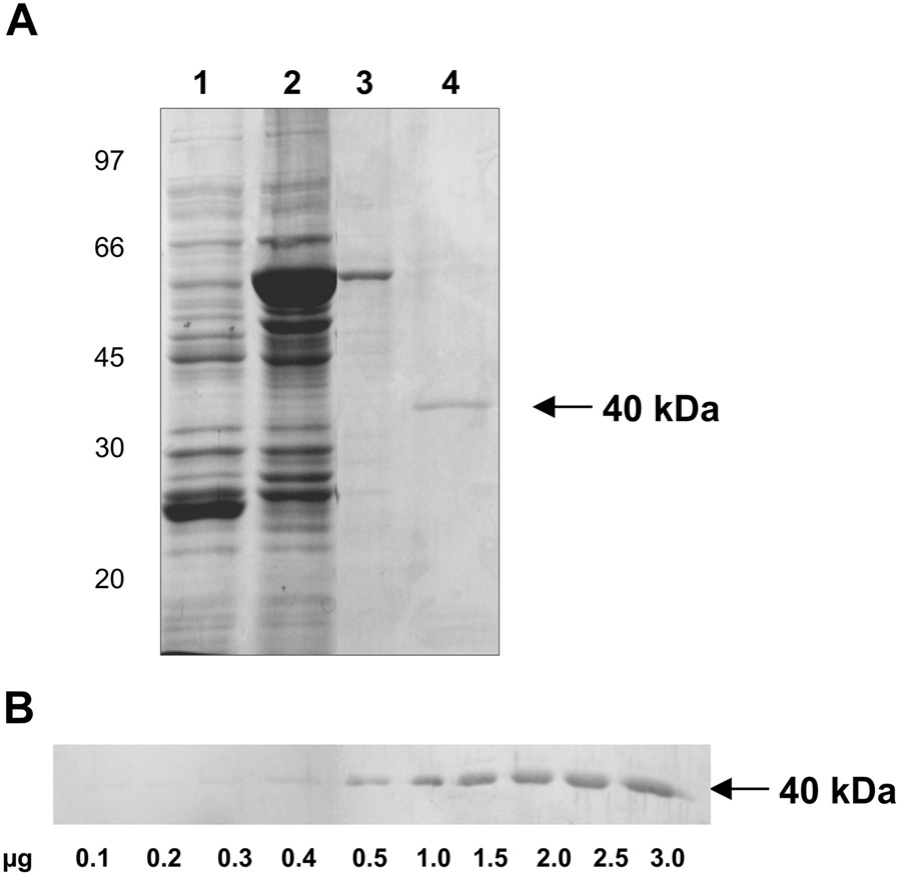
***Paracoccidioides*** **FBA is a plasminogen-binding protein. (A)** SDS-PAGE analysis of the rFBA of *Paracoccidioides*. The recombinant protein was obtained by heterologous expression in *E. coli*. The bacteria total protein extract, before (lane 1), and after (lane 2) the induction with IPTG; the recombinant protein fused to gluthatione S-transferase (GST) (lane 3) and the purified rFBA (lane 4). **(B)** Far-western analysis. Increasing concentrations of the rFBA (0.1 to 3.0 μg) were fractionated by SDS-PAGE (12%) and transfered to a nitrocellulose membrane that was subsequently incubated with hPLg, anti-hPlg antibodies produced in mouse and anti-mouse immunoglobulin G (IgG) coupled to alkaline phosphatase. The reaction was developed using 5-Bromo-4-chloro-3-indolyl phosphate (BCIP) and Nitro Blue Tetrazolium (NBT).

We selected FBA for further analysis, since the protein is a Plg-binding protein in several pathogens, as previously described [15,40,70]. To verify if the FBA of *Paracoccidioides* also has this ability, a recombinant protein was obtained by cloning the cDNA (GenBank Accession Number AY233454) into the expression vector pGEX-4 T-3 (GE Healthcare) as described in Material and Methods. The fusion protein was obtained in *E. coli*. As observed in Figure 2A, the recombinant protein was purified (lane 3) and cleaved from the fusion with GST by the addition of thrombin, rendering a 40-kDa protein (lane 4). A Far-western blotting with increasing concentrations of rFBA was obtained, and depicted in Figure 2B. Concentrations of 0.1 μg to 3 μg of the recombinant protein were subjected to Far-western, demonstrating a dose-dependent binding of the protein with Plg, showing that, in fact, the FBA of *Paracoccidioides* binds to the Plg.

### Detection of FBA at the *Paracoccidioides* surface

**Figure 3:**
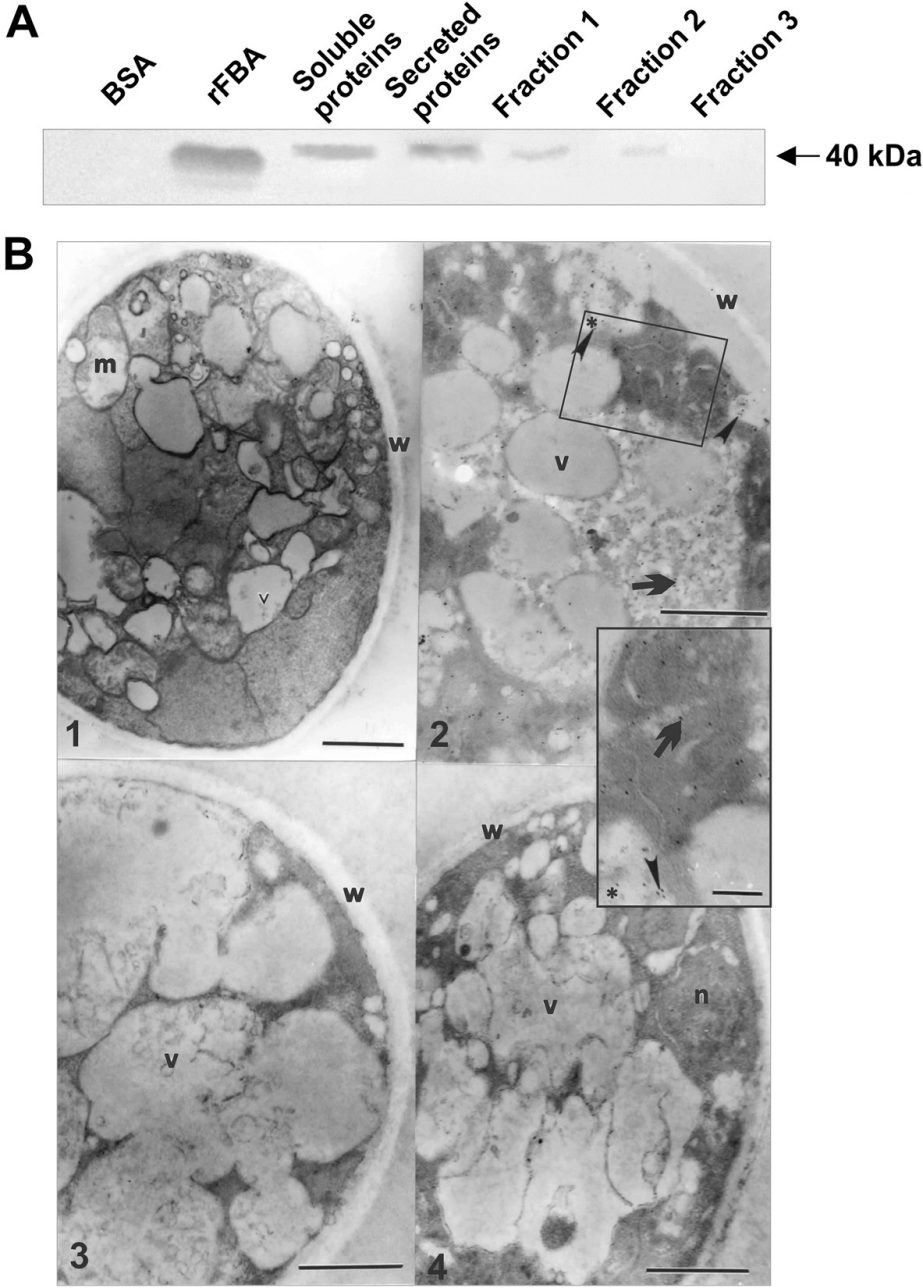
**Detection of FBA in** ***Paracoccidioides*** **. (A)** Western blotting analysis. Different protein samples (15 μg) of *Paracoccidioides* comprehending the soluble and secreted proteins, cell wall fractions 1, 2 and 3 were obtained by sequential treatments as described in Materials and Methods. For negative and positive controls, we employed 3 μg of samples of bovine serum albumin (BSA) and the rFBA, respectively. The immunoblot was probed with the polyclonal antibodies directed to the rFBA. **(B)** Immunoelectron microscopy. Panel 1 - Transmission electron microscopy of *Paracoccidioides* yeast cells showing the cell wall (w), intracytoplasmic vacuoles (v), nucleus (n) and mitochondria (m). Panel 2 - Gold particles are observed in the cytoplasm region (arrows) and vesicles in release process (arrowheads). * corresponds to the region which has been expanded from panel 2. Panels 3 and 4 - Negative controls with anti-rabbit-igG-Au-conjugated and rabbit non immune sera, respectively. The bars indicate: 1.0 μm (Panel 1), 1.0 μm (Panel 2), 0.5 μm (Panel 3), 1.0 μm (Panel 4) and 0.5 μm (Zoom panel).

In order to determine the localization of the FBA in *Paracoccidioides*, *Pb*01, we performed a western blotting with cellular fractions of *Paracoccidioides* and polyclonal antibodies raised in mice to the recombinant protein. As shown in Figure 3A, the FBA is present in the cytoplasm, secretome and cell wall (fractions 1 and 2). The fraction 1 contains proteins associated with the cell surface by non-covalent bonds or by disulfide bridges, as described [71,72]. The fraction 2 represents cell wall proteins sensitive to treatment with alkali (ASL-CWPs), including cell wall proteins with internal repeats (PIR-CWPs). Fraction 3 represents proteins with glycosylphosphatidylinositol (GPI) anchors linked to the wall (GPI-CWPs) [73,74], but rFBA was not detected in this fraction. Furthermore, the immunoelectron microscopy analysis revealed the presence of FBA in the cytoplasm, in vesicles in releasing process and at the cell surface, as depicted in Figure 3B, panel 2. The release of vesicles to the external environment is used by many pathogens to increase their invasive potential. Vesicles contain many virulence factors, including molecules that bind to and activate Plg [27,70,75]. The presence of FBA at the surface and vesicle of the fungus can allow the capture of hPlg and plasmin generation, forming a highly fibrinolytic layer around the fungal cell. These data suggest that FBA, can somehow influence fibrinolytic activity of yeast cells. Cell wall and secreted proteins, may participate in the process.

### *Paracoccidioides* and rFBA bind and activate plasminogen, promoting fibrinolytic activity

**Figure 4:**
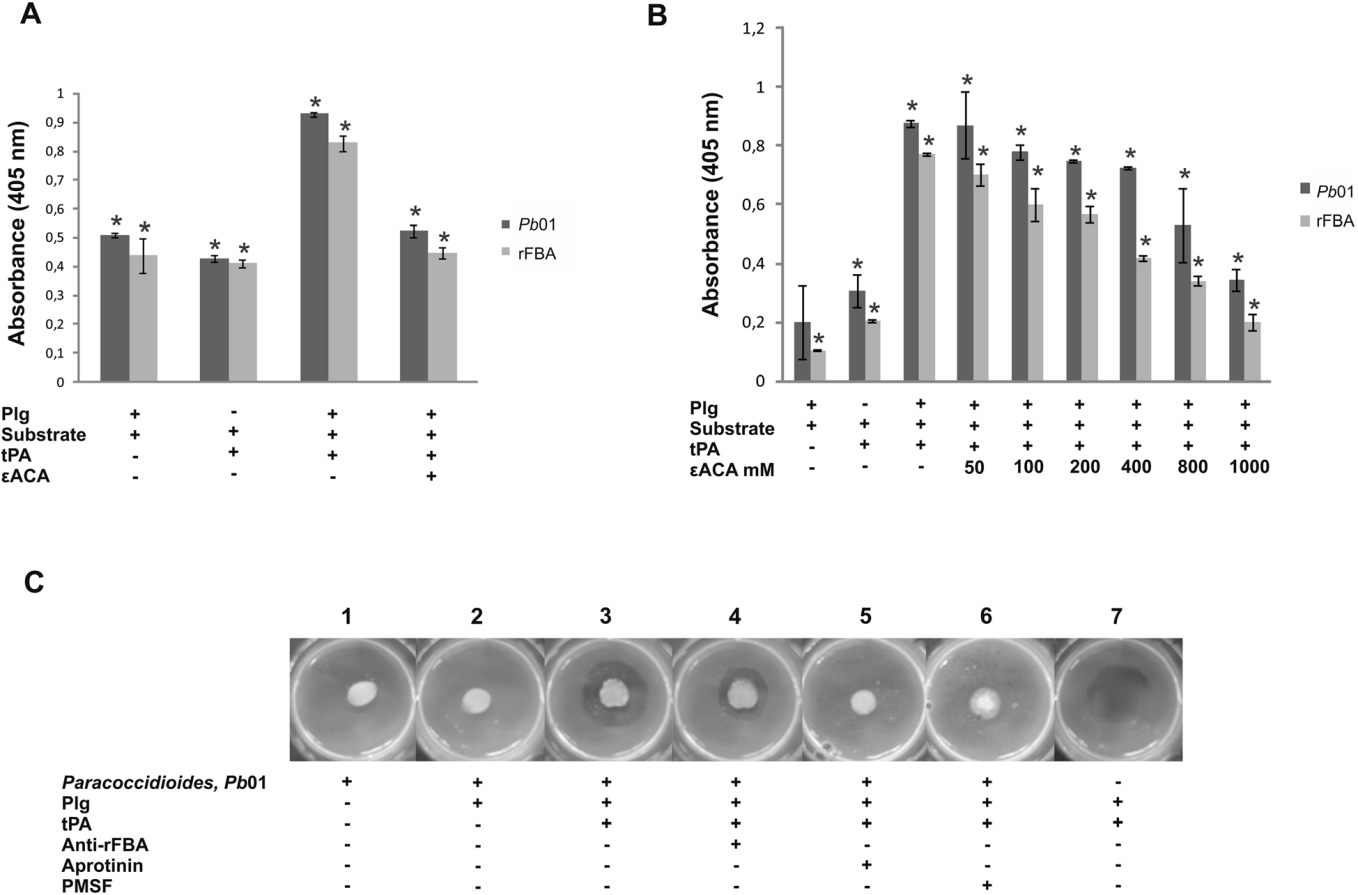
**Plasminogen-binding and activation and fibrin degradation assays. (A)** *Paracoccidioides* yeast cells and the rFBA were incubated with hPlg in the presence or absence of tPA and εACA. We used a plasmin substrate (D-valyl-L-lysyl-p-nitroaniline hydrochloride) (Sigma-Aldrich) to dose the amidolytic activity of the reaction of converting plasminogen into plasmin. **(B)** In competition experiments we added to the wells increasing concentrations of εACA (50 mM to 1 M), followed by the addition of hPlg. Experiments were performed in triplicate as described in Materials and Methods. The error bars indicate the standard deviations between the results. *: results significantly different from control, at a p value < 0.05. **(C)** The fibrinolytic activity of *Paracoccidioides* was analysed by the observation of clear hydrolysis haloes within the opaque jellified-fibrin-containing matrix. Panel 1: *Paracoccidioides* yeast cells in the absence of hPlg; 2: the fungus after binding to hPlg; 3: Similar to 1 and 2, but reflecting the presence of tPA. The fungus was incubated in the presence of hPlg and tPA, with the addition of anti-rFBA (panel 4) and proteases inhibitors, aprotinin and PMSF (panels 5 and 6). Controls consisting of plasminogen and tPA (panel 7).

We next investigated whether the capture of Plg by FBA, favors the generation of plasmin. Previous work from our group have demonstrated that yeast cells of *Paracoccidioides* bind to Plg [10]. As described in Materials and Methods the test was performed by fixation of yeast cells or the rFBA, followed by incubations with hPlg and tPA. In the presence of tPA, the yeast cells and the rFBA were able to generate plasmin. This ability was inhibited by the lysine analogue (εACA), which competes for the binding sites of Plg (Figure 4A). Competition experiments were developed by adding increasing concentrations of εACA, which inhibits plasmin generation in a dose dependent manner (Figure 4B). These data suggest that yeast cells, as well as the recombinant protein bind hPlg, converting into plasmin in the presence of tPA.

Fibrinogen is a major substrate of plasmin *in vivo* and for that, we examined plasmin activity in jellified matrices containing fibrinogen (Figure 4C). Fibrin degradation tests were performed in triplicate (data not shown), where yeast cells were incubated in the presence of hPlg and tPA. It was observed the formation of hydrolysis haloes within the jellified-fibrin-containing matrix (Figure 4C, panel 3). In an attempt to block the receptor of plasminogen on the surface of the fungus, yeast cells were incubated with anti-rFBA polyclonal antibodies (Figure 4C, panel 4). A decrease in the hydrolysis halo comparing the panels 3 to 4, can be observed. The addition of protease inhibitors resulted in no halo formation, due to inactivation of plasmin activity (Figure 4C, panels 5 and 6). Negative controls are presented in panels 1 and 2, whereas positive control is presented in panel 7. Thus, we can conclude that FBA of *Paracoccidioides* may have an important role in the host tissues invasion by the fungus, besides participating in metabolic processes. Corroborating other studies on this subject, the secondary role of this protein makes it an important virulence factor. By capturing and activating Plg, FBA can promote the spread of the fungus, certainly by matrix degradation, paving the way for infection toward internal organs.

### rFBA influences the interaction of *Paracoccidioides* with macrophages

**Figure 5:**
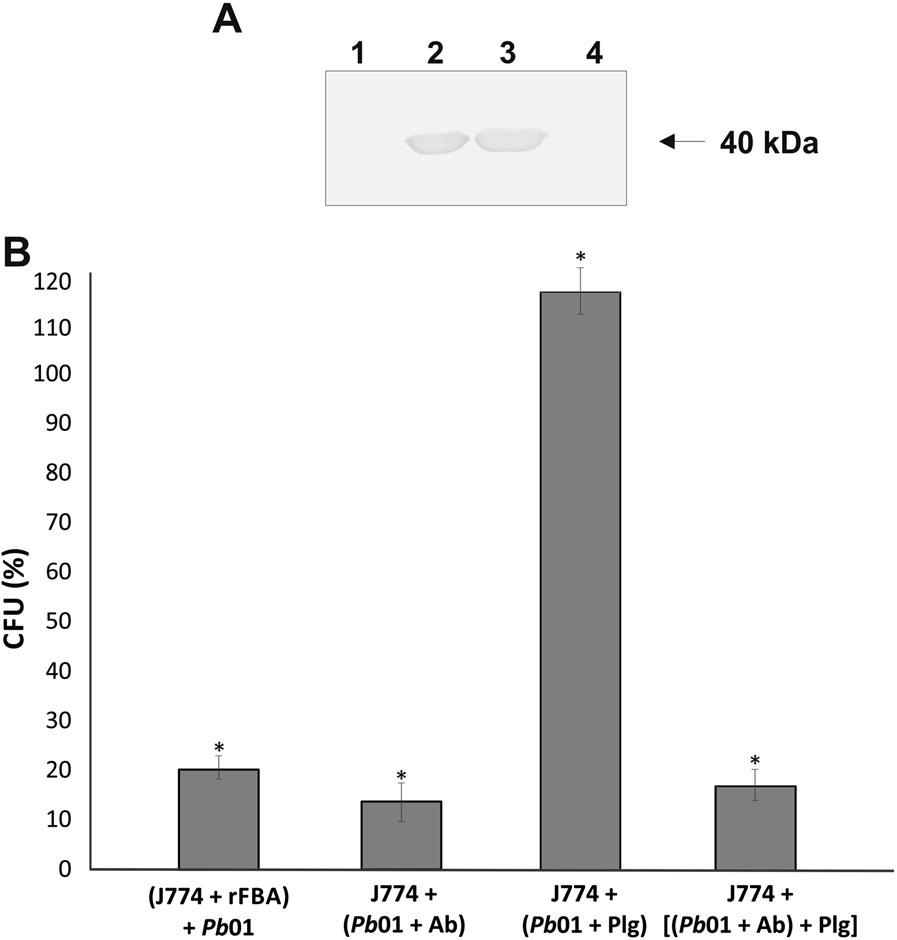
***Paracoccidioides*** **adhesion/internalization by macrophage: Effects of plasminogen and FBA. (A)** Western blotting analysis of binding of J774 macrophages and rFBA. Macrophages were incubated with 50 μg rFBA (line 2). Additionally controls were performed with 5 μg of rFBA for the positive control (line 3), and 5 μg of BSA for negative control (line 4). Line 1 depicts macrophages, without incubation with the rFBA. The immunoblot was probed with the polyclonal antibodies directed to the rFBA. **(B)** Macrophages were or not pre-incubated with the rFBA (50 μg) for 1 h, before infection. Yeast cells (*Pb*01) were or not treated with the antibodies to the rFBA (Ab, 1:1000 diluted), with hPlg (50 μg) and tPA (50 ng), for 1 h, or with the antibodies to the rFBA (Ab, 1:1000 diluted) and subsequently with Plg (50 μg), and tPA (50 ng), for 1 h. Macrophages were incubated with the yeast cells above for 12 h at 36°C. * : results significantly different from control, at a p value < 0.05.

The rFBA of *Paracoccidioides* behaved as an adhesin in a binding assay between J774 and rFBA. Macrophages were able to bind/internalize the rFBA after 5 h incubation (Figure 5A, line 2). Control is depicted in Figure 5A, line 1, in which no reaction was obtained in macrophages not incubated with rFBA. Positive (rFBA, Figure 5A, line 3) and negative (BSA, Figure 5A, line 4) controls, are depicted. Next, we investigated the putative role of FBA in the interaction between *Paracoccidioides* and macrophages. Data represent the percentage of CFUs recovered from infected macrophages, in relation to the control (Figure 5B). The results show that infection of J774 by *Paracoccidioides* was reduced by 79% when the macrophages were pre incubated with rFBA, and 86% when the yeast cells were pre incubated with anti-rFBA antibodies. The data strongly suggest a role for the FBA in the infective process in macrophages.

Similar experiments with other proteins such as glyceraldehyde 3-phosphate dehydrogenase and triose phosphate isomerase, that promoted reduced interaction of *Paracoccidioides* with pneumocytes and Vero cells, were reported [62,76]. Regarding to Plg, the pre-incubation with *Paracoccidioides*, in the presence of tPA, promoted increased macrophage infection (Figure 5B). The addition of the antibodies to rFBA and Plg, prompted inhibited the macrophage infection. This data is consistent with the role of FBA in activating Plg to plasmin, as previously demonstrated in Figure 4C. Our data suggest that binding of the FBA to Plg, may increase the virulence of this pathogen.

## Conclusions

Many microorganisms express proteins that are able to subvert the host proteases and use them in their favor. Once activated to plasmin, Plg acquires fibrinolytic activity. Pathogens able to capture Plg can increase their potential for dissemination in the host tissues. This work identified several secreted proteins of *Paracoccidioides* with ability to bind to hPlg. These proteins are probable targets of the interaction of the fungus with the host, probable acting as mediators of plasmin formation, which may contribute to the invasion of the fungus in the host tissues. The FBA, was detected at the *Paracoccidioides* surface and secretory vesicles, in addition to the conventional cytoplasmatic localization. The protein can bind to hPlg, converting it to plasmin in the presence of tPA. This interaction promoted increased fibrinolytic capacity of the fungus, as demonstrated in fibrin degradation assay. Moreover, we demonstrated that FBA adhered to macrophages and contribute in some way to the interaction of the fungus with these defense cells. These data suggest that FBA is a Plg-binding protein, and may be important virulence factor involved in the process of adhesion, invasion and spread of the fungus.

## Methods

### Strains and media

*Paracoccidioides, Pb*01 (ATCC MYA-826) was used in all experiments. The yeast phase was maintained *in vitro* by sub culturing at 36°C during 7 days in Fava Netto’s solid medium [1% (w/v) peptone, 0.5% (w/v) yeast extract, 0.3% (w/v) proteose peptone, 0.5% (w/v) beef extract, 0.5% (w/v) NaCl, 4% (w/v) glucose, 1.2% (w/v) agar, pH 7.2].

### Preparation of *Paracoccidioides* protein fractions

To obtain the secreted proteins, the yeast cells of *Paracoccidioides, Pb*01 were inoculated in Fava Netto’s liquid medium and cultured at 36°C for 24 h with shaking at 200 rpm, as previously described [48]. The proportion of cells used to obtain the inoculum was 2.5 g wet weight of yeast cells per 50 mL of liquid medium, or 50 mg/mL. After the incubation for 24 h, microscopic analysis was performed to check fungal viability, followed by cells centrifugation at 10,000 × g, for 30 min at 4°C. The supernatant was used for obtain the secreted proteins [48]. The culture supernatant was sequentially filtered through 0.45 mm-pore and 0.22 mm-pore membrane filters. Culture filtrates were concentrated and subsequently washed three times with ultrapure water via centrifugation 10,000 × g through a 10-kDa molecular weight cut off membrane (Amicon ultra centrifugal filter, Millipore, Bedford, MA, USA). The obtained pellet, which contains the yeast cells, was used to the extraction of *Paracoccidioides* soluble [77] and cell wall proteins. Briefly, yeast cells were washed five times with 10 mM Tris–HCl, pH 8.5, 2 mM CaCl_2_ added of the 1:1000 protease inhibitor phenyl methyl sulfonyl fluoride (PMSF) and centrifuged at 10,000 × g for 30 min at 4°C. The cells were frozen in liquid nitrogen and disrupted by maceration. Subsequently, the precipitate was resuspended in lysis buffer (20 mM Tris–HCl pH 8.8; 2 mM CaCl_2_) added of the protease inhibitor PMSF (1:1000) and glass beads; the mixture was agitated for 1 h. After centrifugation 10,000 × g for 30 min at 4°C, the supernatant and pellet were used to obtain the *Paracoccidioides* soluble and cell wall proteins, respectively. The cell wall proteins were extracted by sequential treatments according to the type of connection that these proteins establish with other cell wall components, as previously described, with some modifications [10,71,74,78]. Briefly, the pellet was washed 5 times as following: with cold sterile distilled water, with 5% (w/v) NaCl, with 2% (w/v) NaCl and with 1% (w/v) NaCl. After the washes, the pellet was treated with extraction buffer [50 mM TrisHCl, pH 7.8, 2% (w/v) SDS, 100 mM EDTA and 40 mM β-mercaptoethanol] for 10 min at 100°C. The supernatant from centrifugation constitutes the first fraction (Fraction 1). The pellet resistant to extraction with SDS was washed 5 times with 0.1 M sodium acetate pH 5.5. The obtained solution was centrifuged at 10,000 × g for 30 min at 4°C and the pellet was treated with 30 mM NaOH for 24 h at 4°C, to obtain the second fraction, that after centrifugation at 10,000 × g for 30 min at 4°C, constituted the fraction 2. The pellet was treated with pyridine-hydrogenated fluoride (HF-pyridine) on ice for 24 h to give the third fraction (Fraction 3).

All the obtained protein extracts described above were concentrated and washed three times with ultrapure water via centrifugation through a 10 kDa molecular weight cut off in ultracel regenerated membrane (Amicon ultra centrifugal filter, Millipore, Bedford, MA, USA). The protein concentrations were determined by the Bradford assay using bovine serum albumin as standard [79].

### Two-dimensional gel electrophoresis

Two-dimensional fractionation (2-DE) of secreted proteins was performed, as described [77,80]. The 2-DE gels were obtained in duplicates, using 500 μg of proteins, for each one. The samples were treated with the commercial system of purification 2D Clean-up Kit (GE Healthcare, Uppsala, Sweden) for removing interferences according to the manufacturer’s instructions, before protein isoelectric focusing. Proteins samples were treated with 250 μL of buffer containing 7 M urea, 2 M thiourea, 130 mM 3- [(3-cholamidopropyl) dimethylammonio]-1-propanesulfonate (CHAPS), 0.002% (w/v) dithiothreitol (DTT), ampholyte-containing buffer (IPG buffer, GE Healthcare), and trace amounts of bromophenol blue. Then the samples were loaded onto a 13 cm Immobiline^TM^ DryStrip gel (GE Healthcare) with a pH range of 3–11 for separation of proteins according to their isoelectric points (*pI*) with an electric current of 50 μA / strip at 20°C. In order to perform the first separation of secreted proteins, isoelectric focusing was conducted as following: 30 V for 14 h, 250 V for 1 h (step), 1 kV for 1 h (step), 2 kV for 1 h (step), 5 kV for 3 h (gradient), 8 kV for 8 h (gradient) and 8 kV for 1 h (step). Strips were reduced with 18 mM DTT (dithiothreitol) and alkylated with 135 mM iodoacetamide in equilibration buffer [50 mM Tris–HCl pH 8.8, 6 M urea, 30% (v/v) glycerol, 2% (w/v) sodium dodecyl sulfate (SDS) and 0.002% (w/v) bromophenol blue] during 40 min. The second dimension was performed in 12% polyacrylamide gel electrophoresis under denaturing conditions (SDS-PAGE) in running buffer [25 mM Tris–HCl, 192 mM glycine, 0.1% (w/v) SDS], using a vertical system (GE Healthcare) at 12°C during 1 h at 150 V, and after at 250 V. Two gels were stained by Commassie brilliant blue (Plus One Coomassie Tablets Phast Gel Blue R-350, GE Healthcare) according to manufacturer’s instructions to visualize the proteins.

### Far-western

For the Far-western experiments, the 2-DE gels were produced in duplicates. The secreted proteins, after one or two-dimensional fractionation, were transferred to nitrocellulose membranes for ligand binding with Plg, to identify Plg-binding receptors. The results were compared to the protein pattern of the Coomassie blue stained counterpart. The membranes were incubated in blocking buffer [0.1% (v/v) Tween 20, 5% (w/v) skimmed powder milk, in 10 mM PBS (0.14 M NaCl, 2.7 mM KCl, 10 mM Na_2_HPO_4_, 1.8 mM KH_2_PO_4_, pH 7.3)] for 1 h at room temperature. Subsequently, the membranes were washed three times with PBS containing 0.05% (v/v) Tween 20 (PBS-T) and, except for the negative control, the membranes were incubated with 35 μg/mL of hPlg (Sigma-Aldrich) diluted in blocking buffer, for 1 h under shaking, as described [10]. Subsequently, the membranes were washed three times with PBS-T and incubated with 1 μg/mL anti-human plasminogen (Anti-hPlg) produced in mice (Sigma-Aldrich) diluted 1:100 in blocking buffer. After three washes in PBS-T, the membranes were incubated with the secondary antibody (anti-mouse IgG coupled to alkaline phosphatase) (Sigma-Aldrich) diluted 1:5000 in blocking buffer, for 1 h. After that, the membranes were washed and the reaction was developed using 5-Bromo-4-chloro-3-indolyl phosphate (BCIP) and Nitro Blue Tetrazolium (NBT).

### Expression of the rFBA by *Escherichia coli*, purification of the recombinant protein and polyclonal antibodies production

The cDNA that encodes FBA of *Paracoccidioides, Pb*01 (GenBank Accession Number AY233454) was previously obtained [69]. Oligonucleotide primers were designed: sense S (5’-GAATTCCATGGGCGTGAAAGACA-3’) and antisense AT (5’-GCGGCCGCCTACAACTGGTTAGAA-3’) in order to obtain the cDNA. The cDNA product obtained by RT-PCR was cloned into the expression vector pGEX-4 T-3 (GE Healthcare) and transformed into *Escherichia coli* XL1 blue competent cells. Bacterial cells were grown in Luria-Bertani (LB) medium supplemented with 100 μg/ml ampicillin under agitation at 37°C until the OD reaches an absorbance of 0.6 at a wavelength of 600 nm. The reagent Isopropyl-β-D-thiogalactopyranoside (IPTG) was added to the growing culture to a final concentration of 0.1 mM. After 16 h incubation at 15°C, the bacterial cells were harvested by centrifugation at 10,000 × g for 10 min and resuspended in PBS. Soluble proteins were obtained by sonication, followed by centrifugation at 10,000 × g during 10 min. FBA linked to GST (glutathione-S-transferase) was affinity purified using glutathione Sepharose 4B resin (GE Healthcare). The resin was washed 10 times in PBS and the GST was cleaved by addition of thrombin (50 U/ml) (Sigma-Aldrich). The purity and size of the recombinant protein were assessed by SDS-PAGE followed by staining with Coomassie Blue. The rFBA was used for production of polyclonal antibodies in mice. The purified protein (300 μg) was injected into mice along with Freund’s adjuvant three times at intervals of 15 days. Serum containing polyclonal antibodies was collected and stored at −20°C.

### Western blotting

For western blotting analysis, the *Paracoccidioides* protein samples were probed using polyclonal antibodies produced to the rFBA. Protein samples were loaded onto a 12% SDS-PAGE gel and separated by electrophoresis. The gels were run at 150 V for approximately 2 h and the proteins were transferred to nitrocellulose membranes at 30 V for 16 h in a buffer containing 25 mM Tris–HCl (pH 8.8), 190 mM glycine and 20% (v/v) methanol. The gels were stained with Ponceau red to verify complete protein transfer. Next, each membrane was incubated in blocking buffer [1X PBS, 1.4 mM KH_2_PO_4_, 8 mM Na_2_HPO_4_, 140 mM NaCl, 2.7 mM KCl (pH 7.3), 5% (w/v) nonfat dried milk and 0.1% (v/v) Tween 20] for 2 h. The membranes were washed with PBS-T, and incubated with anti-rFBA polyclonal antibodies (1:1000), followed by washing in blocking buffer three times, during 15 min each wash. The membranes were incubated with the secondary antibody anti-mouse immunoglobulin G (IgG) coupled to alkaline phosphatase (Sigma Aldrich) diluted 1:5000 in blocking buffer, for 1 h. After that, the membranes were washed and the reaction was developed using 5-Bromo-4-chloro-3-indolyl phosphate (BCIP) and Nitro Blue Tetrazolium (NBT).

### Image analyses

The comparative analysis between the images of the proteins stained with Coomassie Blue and the membranes of the Far Western assay were performed using the Image Master 2D Platinum software v7.0 (GE Healthcare) in order to identify in the 2-DE gels the protein spots that were visualized in the membranes through the pairing. The gels and membranes were aligned and the spots were compared according to their isoelectric points and molecular masses.

### Mass spectrometry analysis

The spots of interest were manually excised from the 2-DE gels and treated with trypsin as previously described [48,77,80]. The spots were removed, washed three times with ultrapure water, resuspended in 100 μL of 100% acetonitrile (ACN) and dried in a vacuum centrifuge. Subsequently, the samples were reduced with 10 mM DDT in 25 mM ammonium bicarbonate (NH_4_HCO_3_), and alkylated with 55 mM iodoacetamide in 25 mM NH_4_HCO_3,_ protected from light. The supernatant was removed and the gel pieces were washed with 100 μL of a solution containing 25 mM ammonium bicarbonate/50% ACN (v/v), vortexed for 5 min, and centrifuged. Enzymatic digestion was performed by incubation at 37°C for 16 h in buffer containing trypsin (12.5 ng/μL) and 25 μL of 25 mM NH_4_HCO_3_. The supernatant was transferred to a new tube and the gel pieces were shaken for 30 min in 50% ACN (v/ v), and 1% trifluoroacetic acid (TFA) (v/v), followed by sonication for 5 min, after which the supernatant was combined with the one obtained in the previous step. The dried samples were resuspended in 10 μL of ultrapure water and subsequently purified using a pipette tip with a bed of chromatographic media (ZipTips® C18 Pipette Tips, Millipore, Bedford, MA, USA). Two microliters of each peptide sample were deposited onto a matrix-assisted laser desorption ionization quadrupole time-of-flight mass spectrometry (MALDI-Q-TOF MS) target plate. Next, 2 μL of matrix solution (10 μg/μL a-cyano-4-hydroxyciannamic acid matrix in 50% (v/v) ACN and 5% (v/v) TFA) was added. The mass spectra were performed in the positive reflectron mode on a MALDI-Q-TOF mass spectrometer (SYNAPT, Waters Corporation, Manchester, UK). The MS/MS and PMF analysis was performed using Mascot software v. 2.4 (http://www.matrixscience.com) (Matrix Science, Boston, USA). The ion search parameters were: tryptic peptides with one missed cleavage allowed; fungi taxonomic restrictions; fixed modifications: carbamidomethylation of Cys residues; variable modifications: oxidation of methionine and a tolerance of 0.6 Da. *In silico* analyzes were performed to validate the results obtained *in vitro.* In order to predict the location of proteins we used the program Psort (http://www.genscript.com/psort/wolf_psort.html). The software big-PI Fungal Predictor (http://mendel.imp.ac.at/gpi/fungi_server.html) was used to predict glycosylphosphatidylinositol (GPI) protein anchors. In order to predict proteins to be secreted we employed the Signal P (http://www.cbs.dtu.dk/services/SignalP/) that predicts the classical pathway secretion and Secretome P (http://www.cbs.dtu.dk/services/SecretomeP/) that predicts nonclassical pathway secretion.

### Ultrastructure of the yeast cells and immunocytochemistry of FBA

For the ultrastructural and immunocytochemistry studies, previously described protocols were employed [76,81,82]. The yeast cells were fixed in solution containing 2% (v/v) glutaraldehyde, 2% (w/v) paraformaldehyde, and 3% (w/v) sucrose in 0.1 M sodium cacodylate buffer pH 7.2. Ultrathin sections were stained with 3% (w/v) uranyl acetate and lead citrate. For ultra-structural immunocytochemistry studies, the ultrathin sections were incubated for 1 h with the polyclonal antibodies raised against the rFBA (diluted 1:100) and for 1 h at room temperature with the labeled secondary antibody anti mouse IgG, Au-conjugated (10 nm average particle size; 1:20 dilution). The grids were stained as described above, and observed with a Jeol 1011 transmission electron microscope (Jeol, Tokyo, Japan). Controls were incubated with mouse preimmune serum (1:100 dilution).

### Plasminogen activation assay

The wells of multitier plates were coated with 1 μg of rFBA or fixed with 1 × 10^8^ yeast cells during 1 h. After that, the wells were incubated with 1 μg of hPlg (Sigma-Aldrich), followed by incubation with 3 μg of plasmin substrate (D-valyl-L-lysyl-p-nitroaniline hydrochloride) (Sigma-Aldrich) and 15 ng of tPA (Sigma-Aldrich). Competition and control experiments were performed by blocking the generation of plasmin in the absence of tPA (Sigma-Aldrich) or in the presence of the lysine analogue ε-aminocaproic acid (εACA). The amidolytic activity of the generated plasmin was measured at 405 nm.

### Fibrin matrix-gel degradation analysis

The matrix gel contained 1.25% (w/v) low-melting-temperature agarose, 100 μg of hPlg (Sigma- Aldrich) and 4 mg of fibrinogen (Sigma- Aldrich) in a final volume of 2 mL. To detect fibrinolysis activity, a total 1 × 10^7^ cells of *Paracoccidioides, Pb*01 were incubated with 50 μg of hPlg for 3 h in the presence or absence of tPA (50 ng). The yeast cells were also incubated with the serine proteinase inhibitors aprotinin (1 μg), PMSF (50 mM) and with anti-rFBA antibodies in a final volume of 1 mL. Thereafter, the mixtures were washed three times with PBS and the pellets were placed in wells of a fibrin substrate matrix gel. Plasmin activity was detected by the observation of clear hydrolysis haloes within the opaque jellified fibrin-containing matrix, after incubation in a humidified chamber at 37°C for 12 h.

### Binding assays of the rFBA to *in vitro* cultured macrophages

J774 1.6 macrophages (Rio de Janeiro Cell Bank – BCRJ/ UFRJ, accession number 0273) were used for phagocytosis assays. The J774 1.6 cells were cultured in RPMI medium containing bovine fetal serum 10% (v/v) (Vitrocell Embriolife,) containing IFN-γ (1U per mL) and MEM non-essential amino acid solution (Sigma Aldrich, Missouri, USA) at 36°C and 5% CO_2_, until complete confluence. The macrophages were incubated with 50 μg/mL of rFBA, at 36°C for 5 h, and washed. Next, the cells were lysed by incubating with distilled water for 1 h. The lysate was centrifuged at 1,400 × g for 5 min. The proteins contained in the supernatant were submitted to SDS-PAGE and transferred to nitrocellulose membrane. The membrane was incubated blocking buffer [PBS 1X with 5% (w/v) nonfat dried milk and 0.1% (v/v) Tween 20] for 2 h, and then successively with anti-rFBA polyclonal antibodies (1:1000) and with the anti-mouse immunoglobulin G (IgG) coupled to alkaline phosphatase (Sigma Aldrich). The reactions were developed with BCIP-NBT.

### rFBA and anti rFBA-antibodies decrease *Paracoccidioides* macrophages interaction

We tested the interference of the rFBA and antibodies to adhesion/infection of *Paracoccidioides* in macrophages. In addition, we tested the ability of Plg-treated yeast cells to adhere/infect macrophages. A total of 5 × 10^6^ yeast cells, per well, were added to the macrophages, reaching a yeast:macrophages cells ratio of 5:1, followed by incubation for 12 h at 36°C, in 5% CO_2,_ in RPMI medium containing IFN-γ (1U per mL). The J774 cells were pre incubated for 1 h at 36°C with the rFBA (50 μg/ml), or the yeast cells were pre- incubated with anti-rFBA antibody (1:1000) and then the infection was performed. In parallel, yeast cells were incubated with the polyclonal antibody anti-rFBA (1:1000 diluted) for 1 h at 36°C, and then incubated with plasminogen (50 μg) and tPA (50 ng) for 1 h at 36°C. After that, the yeast cells were washed three times in PBS 1X and incubated with the macrophages.

At the end of the infection, the adhered macrophages were washed, lysed by addition of distilled water and centrifuged. The pellet was diluted 1:10 and plated in solid BHI medium supplemented with inactivated fetal calf serum (4% v/v). After 7 days at 37°C the number of CFU’s was counted.

### Statistical analysis

The experiments were performed in triplicate, with samples in triplicates. Results are presented as means ± standard deviations. Statistical comparisons were performed using Student’s *t* test and the statistical significance was accepted for *P* value of < 0,05.
